# Expansion and Antitumor Cytotoxicity of T-Cells Are Augmented by Substrate-Bound CCL21 and Intercellular Adhesion Molecule 1

**DOI:** 10.3389/fimmu.2018.01303

**Published:** 2018-06-11

**Authors:** Shimrit Adutler-Lieber, Nir Friedman, Benjamin Geiger

**Affiliations:** ^1^Department of Molecular Cell Biology, Weizmann Institute of Science, Rehovot, Israel; ^2^Department of Immunology, Weizmann Institute of Science, Rehovot, Israel

**Keywords:** CCL21, intercellular adhesion molecule 1, T-cell immunity, cancer immunotherapy, T-cell cytotoxicity, T-cell clusters

## Abstract

Adoptive immunotherapy is based on *ex vivo* expansion and stimulation of T-cells, followed by their transfer into patients. The need for the *ex vivo* culturing step provides opportunities for modulating the properties of transferred T-cells, enhancing their antitumor abilities, and increasing their number. Here, we present a synthetic immune niche (SIN) that increases the number and antitumor activity of cytotoxic CD8^+^ T-cells. We first evaluated the effect of various SIN compositions that mimic the physiological microenvironment encountered by T-cells during their activation and expansion in the lymph node. We found that substrates coated with the chemokine CCL21 together with the adhesion molecule intercellular adhesion molecule 1 significantly increase the number of ovalbumin-specific murine CD8^+^ T-cells activated by antigen-loaded dendritic cells or activation microbeads. Notably, cells cultured on these substrates also displayed augmented cytotoxic activity toward ovalbumin-expressing melanoma cells, both in culture and *in vivo*. This increase in specific cytotoxic activity was associated with a major increase in the cellular levels of the killing-mediator granzyme B. Our results suggest that this SIN may be used for generating T-cells with augmented cytotoxic function, for use in cancer immunotherapy.

## Introduction

Adoptive immunotherapy is broadly considered a promising approach for induction of antitumor immune responses, based on the isolation of specific T-cells, their *ex vivo* activation or genetic manipulation, expansion, and subsequent autologous administration (1–4). Despite its great potential, this emerging field still presents major challenges (5, 6). A critical limiting factor is the need to selectively expand tumor-specific T-cells in high quantities, sufficient for effective tumor eradication or suppression. In addition, the desired activity of the expanded cells must be maintained, overcoming the common tendency of proliferating T cell populations to develop impaired functionality due to anergy (7), exhaustion (8, 9), and suppressing signals that stem from stromal cells (10, 11), other immune cells (12), or from the tumor itself (13, 14). These limitations prompted us to search for conditions that would improve the expansion, cytotoxicity, and antitumor activity of CD8^+^ T-cells, through design of a suitable synthetic immune niche (SIN).

During an immune response, T cell activation involves complex sets of cellular interactions and paracrine stimulations, all of which take place at specific sites within the lymphatic system, commonly referred to as “immune niches” (15–18). Mimicry of such niches by engineering SINs is an emerging field, with important implications for adoptive therapies (2, 4, 19, 20). To function effectively, a SIN should encompass the broad diversity of natural immune niches, and enable the complex interplay between the different cell types that reside within them. Several SIN engineering approaches, based on various geometries, with different physical and chemical parameters (2, 21–27), have produced valuable insights into the molecular complexity of the corresponding immune responses. Yet, limited knowledge exists concerning the synergy between different SIN parameters, their signaling specificity, as well as the role of topology in their effective integration.

The aforementioned considerations motivated us to study novel SIN designs, with antigen-mediated activation of T-cells on substrates coated with chemokines and adhesion molecules, in the presence of different cytokines. The choice of specific molecules for the design of the SIN was based on known molecular components of the lymphatic niche, which facilitate the interactions of T-cells with antigen-presenting cells, and promote the activation, expansion, and proliferation of antigen-specific T-cells. Selected molecules were experimentally tested, both alone and in combination, for their effects on the cultured T-cells.

We recently demonstrated that a combination of three such factors; namely, the chemokine CCL21, the intercellular adhesion molecule 1 (ICAM1), and the cytokine IL-6, results in a SIN that augments the expansion and survival of CD4^+^ T-cells (28). CCL21, secreted by lymphatic stroma and endothelial cells (29), induces several processes critical to immune responses: co-localization and recruitment of T-cells and dendritic cells (DCs) (30, 31); improved cell migration (25, 32); priming of T-cells for synapse formation (33); and co-stimulation of naïve T-cell expansion and Th1 polarization (27, 34, 35). ICAM1 plays a key role in the formation of immune synapses and promotion of T cell activation, through binding to its integrin ligand, lymphocyte function-associated antigen 1 (LFA1) (36, 37). These factors act synergistically, as CCL21 increases LFA1 responsiveness to ICAM1, and mediates the arrest of motile lymphocytes on ICAM1-expressing DCs and endothelial cells, and also their clustering with other T-cells (22, 29). Finally, the cytokine IL-6 is secreted by the lymphatic stroma to support T-cell survival (38, 39).

In the present study, we tested whether the SIN described above can stimulate the expansion of cytotoxic CD8^+^ T-cells. Moreover, we further examined the effect of this treatment on the cytotoxic activity of the cultured cells. Here, we report that substrate-bound CCL21 and ICAM1 not only increase the yield of CD8^+^ T-cells but also elevate the cellular levels of granzyme B expressed in these cells. Consequently, T-cells cultured on CCL21 + ICAM1-coated substrates exhibited an improved killing of cultured cancer cells, and markedly elevated tumor suppressive activity, *in vivo*.

## Materials and Methods

### Mice

C57BL/6 mice were obtained from Harlan Laboratories (Rehovot, Israel), and OT-I mice (40) from Jackson Laboratories (Bar Harbor, ME, USA). All mice were 5–12 weeks old, were maintained at the Weizmann Institute’s Lorry Lokey Pre-Clinical Research Facility, and cared for in accordance with national and institutional guidelines. Experiments were approved by the Institutional Animal Care and Use Committee.

### Primary Mouse Cell Isolation and Culture Handling

CD8^+^ T-cells were purified (>95%) from a cell suspension harvested from crushed spleens of OT-I mice, using a CD8a^+^ T Cell Isolation Kit and magnetic-associated cell sorting (MACS), according to the manufacturer’s instructions (Miltenyi Biotec, Bergisch Gladbach, Germany). Similarly, DCs were purified (>85%) from spleens of C57BL/6 mice, using MACS CD11c microbeads (Miltenyi Biotec). T-cells and DCs were cultured immediately following isolation in a ratio of 3:1, respectively, with 1 µg/ml of ovalbumin peptide (OVA257-264, InvivoGen, San Diego, CA, USA) in a RPMI 1640 medium w/o phenol red, supplemented with 10% serum, 100 U/ml of penicillin, 100 mg/ml of streptomycin, 2 mM glutamine, 10 mM HEPES, 1 mM sodium pyruvate, and 50 mM β-mercaptoethanol (Biological Industries, Beit Haemek, Israel).

### Substrate Functionalization and Soluble Factors

Substrate functionalization was performed by overnight incubation in PBS with 5 µg/ml CCL21 and/or 50 µg/ml ICAM1 (R&D Systems, Minneapolis, MN, USA).

### Microscopy and Image Analysis

Time-lapse movies were acquired using a DeltaVision Elite^®^ microscope (Applied Precision, GE Healthcare, Issaquah, WA, USA) mounted on an inverted IX71 microscope (Olympus, Center Valley, PA, USA) connected to a Photometrics CoolSNAP HQ2 camera (Roper Scientific, Martinsried, Germany). The primary image processing software used was SoftWorX 6.0. This microscope was also used for the production of deconvolution-based 3D image reconstructions, renderings supported by BITPlan software (Willich-Schiefbahn, Germany). Wide-range phase-contrast images and high content/high throughput microscopy was conducted on 96-well plates using a Hermes^®^ microscope (IDEA BioMedical Ltd., Rehovot, Israel) equipped with automated scanning optics, high-precision autofocus, and a closed environmental chamber.

### Cell Viability Assay and Enumeration

A microscopic cell-counting assay was performed using automated analysis of fluorescent microscope images. Live cells were identified by staining the cell nuclei with 1 µg/ml Hoechst (33342, ImmunoChemistry Technologies, Bloomington, MN, USA) and with 250 ng/ml propidium iodide (Sigma Aldrich, St. Louis, MO, USA), for elimination of dead cells from the analysis. At the experimental end-point, cells were pipetted in order to break up cell clusters, then spun down. Fluorescence images were taken using a Hermes microscope (IDEA Bio-Medical Ltd.) and image analysis software (WiSoft, IDEA Bio-Medical Ltd.) was used to quantify viable cell numbers.

A metabolic viability assay was performed by adding 20 µl CellTiter-Blue (Promega Corporation, Madison, WI, USA) per 100 µl culture medium for 3 h. Results were quantified using a fluorescence plate reader (excitation 560 nm; emission 590 nm), and a linear equation of a calibration column.

### Cell Proliferation Assay

T-cells were stained prior to seeding with 5 µM CFSE (Biolegend, San Diego, CA, USA) for 20 min at 37°C, according to the manufacturer’s instructions. Excess dye was removed by washing with five volumes of RPMI. T-cells were seeded with DCs: 30–60 × 10^3^:10–20 × 10^3^, respectively, with 1 µg/ml ovalbumin peptide (OVA257-264, InvivoGen, San Diego, CA, USA) in a 96-well plate with 250 µl complete RPMI medium. Five days later, cells were detached from the substrate using 10 min incubation with PBS without calcium and magnesium, and pipetting. Propidium iodide (1 µg/ml) was added to each well for cell death staining. Single-cell suspensions were taken for flow cytometry analysis (Becton Dickinson, Franklin Lakes, NJ, USA); and FlowJo software (Ashland, OR, USA). Since CFSE is diluted by approximately half with each cell division, live single cells (negative PI staining) were gated, and the mean fluorescence intensity of their CFSE was used to evaluate their level of proliferation.

### Scanning Electron Microscopy

T-cells were activated and cultured with or without substrate coatings for 7–9 days, harvested, and counted. Target cells were seeded on glass coverslips placed inside wells of a 24-well plate (250,000/well) with the harvested T-cells (750,000/well) for 16–24 h. Wells were gently washed in 0.1 M cacodylate buffer, fixed with Karnovsky fixative (2% glutaraldehyde, 3% PFA, in 0.1 M cacodylate buffer), and incubated overnight at 4°C. Cover glasses were dehydrated in increasing concentrations of ethanol (30, 50, 70, 96, and 100%), followed by critical-point drying in BAL-TEC CPD030, and sputtering in a gold palladium sputter coater (Edwards, Crawley, UK). Images were taken using a secondary electron (SE) detector in a high-resolution Ultra 55 scanning electron microscope (Zeiss, Oberkochen, Germany).

### *In Vitro* Cytotoxic Murine T-Cell Killing Assay

B16 cells expressing ovalbumin coupled with GFP (courtesy of Guy Shakhar, Weizmann institute of Science) were suspended in RPMI 1640 medium w/o phenol red, supplemented with 10% serum, 100 U/ml of penicillin, 100 mg/ml of streptomycin, 2 mM glutamine, 10 mM HEPES, 1 mM sodium pyruvate, and 50 mM β-mercaptoethanol (Biological Industries). Cells were seeded in a 384-well plate, 1,000 cells per well, and incubated for 2–3 h, to enable their attachment to the substrate. OT-I T-cells activated and cultured for 3 or 7 days as described above, were then added on top of the B16 cells. The entire well was imaged every 6 h, using a Hermes^®^ microscope (IDEA BioMedical Ltd.) equipped with automated scanning optics, high-precision autofocus, and a closed environmental chamber. The number of live (e.g., GFP expressing) B16 cells was then counted from the image, using WiSoft^®^ software (IDEA Bio-Medical Ltd.).

### Measuring Granzyme B, FasL, and PD-1 Expression Level

B16 cells-expressing ovalbumin (courtesy of Lea Eisenach, Weizmann institute of Science) were suspended in DMEM medium w/o phenol red, supplemented with 10% fetal bovine serum, 100 U/ml of penicillin, 100 mg/ml of streptomycin, 2 mM glutamine, 10 mM HEPES, 1 mM sodium pyruvate, and 50 mM β-mercaptoethanol (Biological Industries). Cells were seeded 50–100 × 10^3^ B16 cells per well in a 24-well plate; 150–300 × 10^3^ OT-I T-cells pre-activated and cultured for 7 days, were added on top of the B16 cells. After 24–48 h, cells were collected, and a single-cell suspension was prepared and stained with live/dead stain (L23105, Life Technologies, Carlsbad, CA, USA), CD8 (BLG100734, Biolegend), FasL (BLG106606, Biolegend), and PD-1 (BLG135225, Biolegend). Cells were then fixated (BLG420801, Biolegend), permeablized (BLG521002, Biolegend), and stained for intracellular granzyme B (BLG515406, BioLegend). The mean fluorescent intensity of Granzyme B, FasL, and PD-1 of live CD8^+^ cells was measured and analyzed using flow cytometry (Becton Dickinson; FlowJo software).

### *In Vivo* Cytotoxic T-Cells Tumor Suppression Assay

B16 melanoma cell line expressing ovalbumin (courtesy of Lea Eisenbach, Weizmann Institute of Science) were grown in DMEM medium, supplemented with 10% serum, 100 U/ml of penicillin, 100 mg/ml of streptomycin, 2 mM glutamine, 10 mM HEPES, 1 mM sodium pyruvate, and 50 mM β-mercaptoethanol (Biological Industries). Cells were harvested using trypsin, washed twice, and suspended in PBS (Biological Industries). For each C57BL/6 mouse, 50 µl of cell suspension containing 2 × 10^6^ cells were injected orthotopically into the flank skin. Seven days following the injection of the tumor cells, tumor size was documented by measuring with a caliper, the two longest vertical diameters of the tumor. Multiplication of these diameters was used to exclude mice bearing tumors with dimensions smaller than 15 mm^2^, or larger than 35 mm^2^. The rest were split into groups with similar averages and size distributions. OT-I T-cells activated and cultured for 7 days as described above were washed twice and suspended in PBS. For each tumor-bearing C57BL/6 mouse, 100 µl containing 2 × 10^6^ or 4 × 10^6^ T-cells were injected intravenously. Three days following T-cell injection, and every 2–3 days subsequently, tumor size was documented by measuring, with a caliper, the two longest vertical diameters of the tumor. The average of these two diameters (*D*) was then used to calculate the volume of a ball with the following formula: 4/3 × π × (*D*/2)^3^.

### T-Cell Activation With Activation Beads

Freshly isolated CD8^+^ T-cells were incubated 1:1 with activation beads freshly prepared according to the manufacturer’s instructions (Miltenyi Biotec) and IL-2, 30 U/ml (BioLegend).

### Statistical Analysis

Data shown in each graph constitute an average of 3–10 replicates that were cultured and measured separately in a single independent experiment, representative of more than three separate, repeated experiments. Error bars indicate SEM, and statistical significance was calculated using a standard *t*-test.

## Results

### Substrate-Immobilized CCL21 Increases the Size of CD8^+^ T-Cell Clusters, While Substrate-Immobilized ICAM1 Transforms T-Cell Clusters Into Substrate-Attached Monolayers

As a model for antigen-specific cytotoxicty, we used CD8^+^ T-cells isolated from the spleens of OT-I mice, which express an ovalbumin-specific T cell receptor (TCR). These cells were expanded *ex vivo* by co-culturing with ovalbumin-loaded DCs serving as antigen-presenting cells.

**Figure 1:**
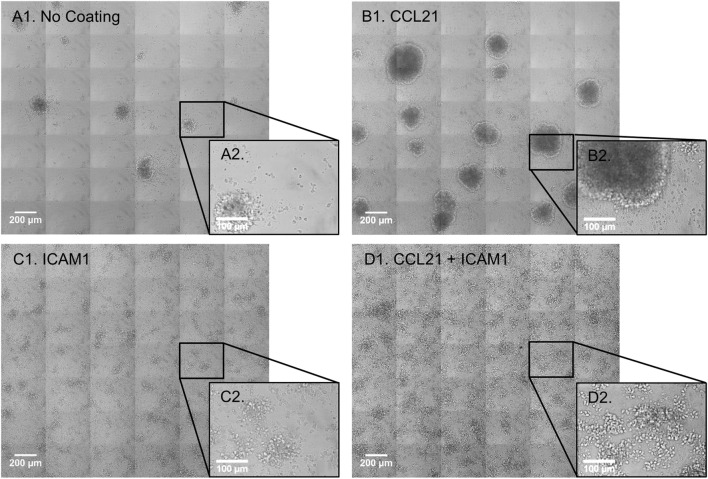
Substrate-immobilized CCL21 increases the size of CD8^+^ T-cell clusters, while substrate-immobilized intercellular adhesion molecule 1 (ICAM1) transforms the clusters into substrate-attached monolayers. **(A1–D2)** Transmitted light microscopy images depicting morphological changes in co-cultures of OT-I CD8^+^ T-cells and ovalbumin-loaded dendritic cells (DCs). Primary T-cells and DCs were isolated from spleen and were immediately co-cultured for 72 h, on either an uncoated substrate **(A1,A2)**, substrate-immobilized CCL21 **(B1,B2)**, substrate-immobilized ICAM1 **(C1,C2)**, or substrate-immobilized CCL21 + ICAM1 **(D1,D2)**. Substrate-immobilized CCL21 **(B1,B2)** induced larger T-cell clusters compared to the uncoated substrate **(A1,A2)**, while ICAM1 alone **(C1,C2)**, or in combination with CCL21 **(D1,D2)**, induced cell spreading and decreased cluster size. Scale bar in **A1**, **B1**, **C1**, and **D1**: 200 µm. Black squares **(A2–D2)** show enlarged regions, with a scale bar of 100 µm.

We began by evaluating the effect of substrate-attached CCL21 and ICAM1 on the morphology of the cultured T-cells. Substrate-attached CCL21 (Figures 1B1,B2) induced the formation of more T-cell clusters (53 clusters per 5.1 mm^2^) with a larger projected area (0.068 mm^2^ on average, range: 0.005–0.332 mm^2^), compared to those plated on an uncoated substrate (Figures 1A1,A2; 17 clusters per 5.1 mm^2^, with an average projected area of 0.046 mm^2^, range: 0.003–0.101 mm^2^). On the other hand, CD8^+^ T-cells seeded on a substrate coated with ICAM1 alone (Figures 1C1,C2) or on a CCL21 + ICAM1-coated substrate (Figures 1D1,D2) formed 2D monolayers of mostly spread cells. These findings show that the effects of CCL21 and ICAM1 on culture morphology are similar for both CD8^+^ T-cells (shown here) and CD4^+^ T-cells, as we previously showed (28).

### Substrate-Immobilized CCL21 + ICAM1 Collectively Augment CD8^+^ T-Cell Expansion

**Figure 2:**
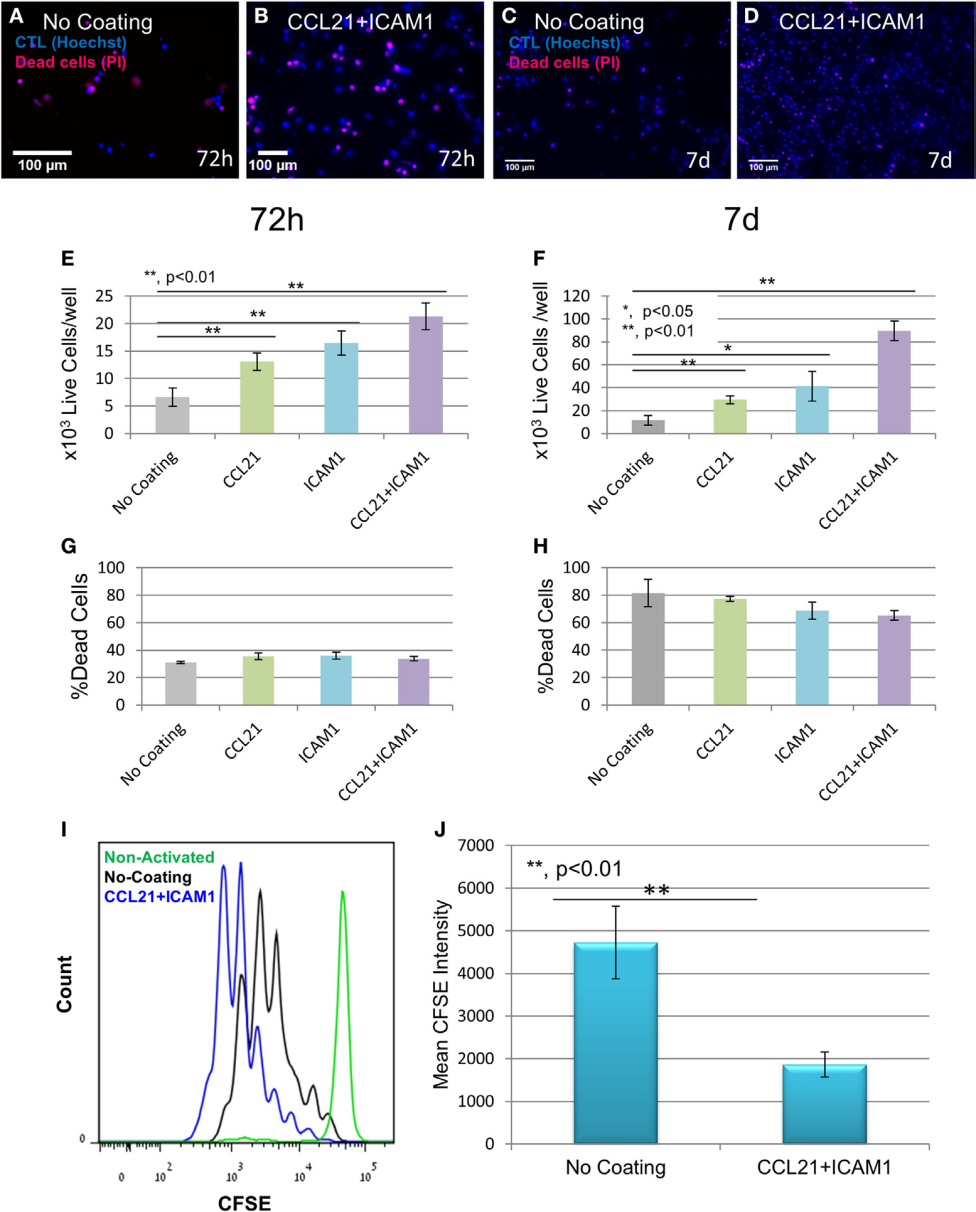
Substrate-immobilized CCL21 + intercellular adhesion molecule 1 (ICAM1) increase cytotoxic T-cell number and proliferation. **(A–D)** Representative fluorescence images of T-cells grown on different coated substrates for 72 h **(A,B)** or 7 days **(C,D)**, following the breakdown of cell clusters, and their spin-down. Cell nuclei are stained blue in all cells, and red only in dead cells. Scale bar: 50 µm. **(E–H)** Viable cell numbers and percentage of dead cells at 72 h (**E,G**, respectively) and 7 days (**F,H**, respectively), quantified using automated image analysis. Data are from one experiment representative of at least three independent experiments with 20 replicates each (see Figure S1A in Supplementary Material). Error bars represent SEM. Calculated *p*-values (using standard *t*-test) are as indicated in the Figure. The number of T-cells seeded per well was 3 × 10^3^. CCL21 and ICAM1 coatings collectively increase viable cell numbers by up to ninefold, without significantly affecting cell death. **(I,J)** Histogram and bar graph illustrating the increase in cell proliferation induced by CCL21 + ICAM1, indicated by a decrease in the mean fluorescent intensity of CFSE, compared to cells grown on the uncoated culture (data are representative of three independent experiments with four replicates each). Error bars represent SEM. Calculated *p*-values (using standard *t*-test) are as indicated in the Figure]. Histograms in **(I)** show CFSE levels: green—non-activated T-cells; black —T-cells activated on uncoated substrates; blue—T-cells activated on CCL21 + ICAM1-coated substrates.

Substrate-immobilized CCL21 + ICAM1 not only altered the cellular organization and culture morphology of CD8^+^ T-cells but also affected T-cell yield. We quantified this effect by labeling the cell nuclei with Hoechst (which stains nuclei in all cells) and propidium iodide (which stains only dead cells), followed by automated image analysis. Representative images (Figures 2A–D), as well as image-based quantification of viable cell numbers (Figures 2E,F; Figure S1A in Supplementary Material) revealed that substrate-immobilized CCL21 + ICAM1 substantially increased the number of viable CD8^+^ T-cells compared to controls, but had only a marginal effect on the fraction of dead cells (Figures 2G,H). We note the relatively high level of cell death observed at 7 days of culture for all conditions, which can result from prolonged antigen stimulation. After 7 days of culture, the CCL21 + ICAM1-coated substrates induced a ninefold increase in the number of viable cells, compared to the uncoated substrate (Figure 2F). These results are similar to the combined effects of CCL21 + ICAM1 on CD4^+^ T-cells (28). We further evaluated the effect of CCL21 + ICAM1 on T-cell proliferation. CFSE staining of cells prior to seeding, followed by flow cytometry analysis 5 days later, showed that the contribution of CCL21 + ICAM1 to the elevated cell yield was due to increased T-cell proliferation (Figures 2I,J; Figure S1B in Supplementary Material).

**Figure 3:**
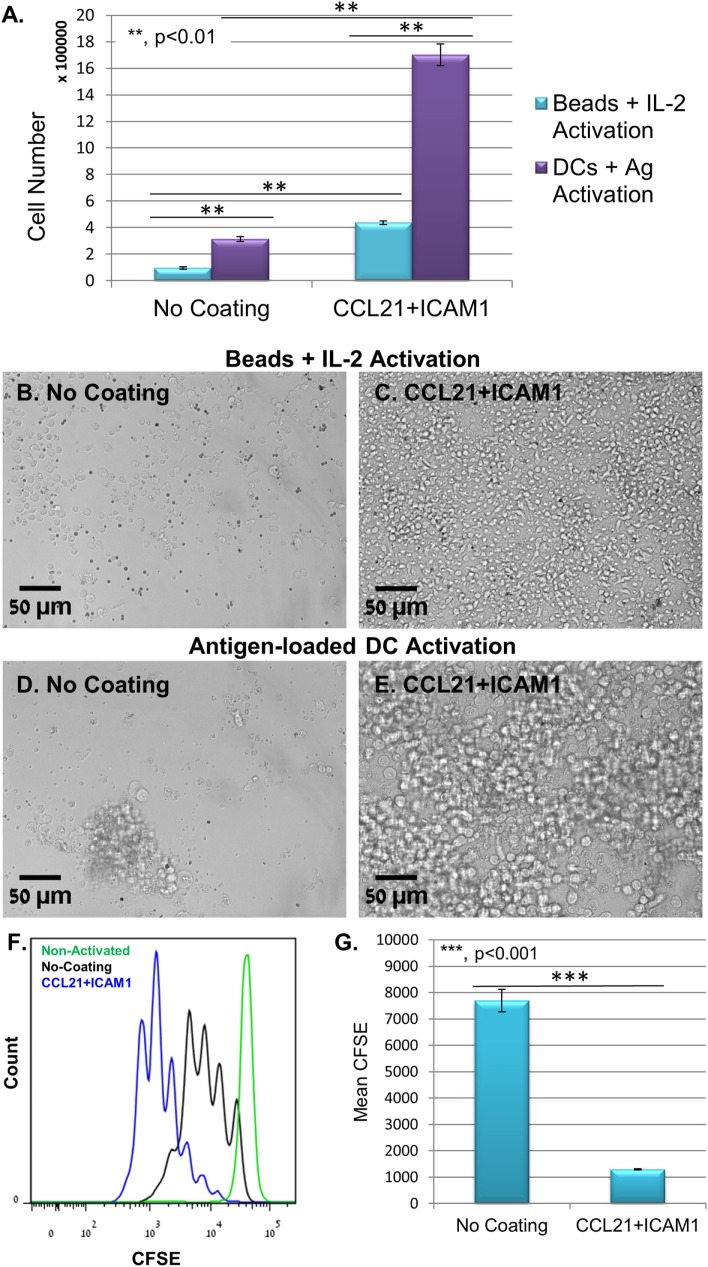
Coating with CCL21 + intercellular adhesion molecule 1 (ICAM1) increases proliferation of T-cells activated with αCD3/αCD28 microbeads, but with a smaller cell yield, compared to their activation with antigen-loaded dendritic cells (DCs). **(A)** Bar graph illustrating live cell number, measured using a metabolic cell viability assay of T-cells activated with either antigen-loaded DCs or activation microbeads, with or without CCL21 + ICAM1 substrate coating. Data are representative of at least three independent experiments with five replicates each. Error bars represent SEM. Calculated *p*-values (using standard *t*-test) are as indicated in the Figure. The number of T-cells seeded per well was 30 × 10^3^. **(B–E)** Representative images demonstrating the higher cell density in cultures with CCL21 + ICAM1 compared to no coating, activated with either activation beads **(B,C)** or antigen-loaded DCs **(D,E)** for 5 days. Scale bars: 50 µm. **(F,G)** Histogram and bar graph illustrating the increase in cell proliferation induced by CCL21 + ICAM1, indicated by a decrease of 6.5-fold in the mean fluorescent intensity of CFSE, compared to cells cultured on uncoated substrates [data are from one experiment representative of three independent experiments with four replicates each (see Figure S1B in Supplementary Material). Error bars represent SEM. Calculated *p*-values (using standard *t*-test) are as indicated in the Figure]. Histograms in **(F)** show CFSE levels: green—non-activated T-cells; black—T-cells activated on uncoated substrates; blue—T-cells activated on substrates coated with CCL21 + ICAM1.

Substrate-immobilized CCL21 + ICAM1 affected also CD8^+^ T-cells that were activated using anti-CD3/CD28-coated microbeads and IL-2. We found that non-specific activation with the beads yielded significantly lower numbers of T-cells, compared to antigen-specific activation with DCs (Figures 3A–E). Regardless, the CCL21 + ICAM1 coating significantly augmented T-cell proliferation following activation with beads (Figures 3F,G; Figure S1B in Supplementary Material), similar to its effect on proliferation following activation with antigen-loaded DCs (Figures 2I,J; Figure S1B in Supplementary Material).

Notably, the positive effect of substrate-immobilized CCL21 + ICAM1 on T-cell expansion (compared to control) was conspicuous even when the number of seeded CD8^+^ T-cells was extremely low (<1,000 cells; see Figure S2 in Supplementary Material). For instance, when seeding 750 cells per uncoated well of a 96-well plate (~25 cells/mm^2^), only a few small clusters were formed. Seeding the same number of cells in wells functionalized with CCL21 + ICAM1 resulted in the formation of dense cultures, with a few large clusters (see Figure S2B in Supplementary Material).

### Substrate-Immobilized CCL21 + ICAM1 Augment the Killing Capacity of Ovalbumin-Specific CD8^+^ T-Cells Toward Ovalbumin-Expressing Melanoma Cells

Given that the primary objective of this study was to use the SIN for generating expanded populations of functional T-cells, we tested the effect of substrate-immobilized CCL21 + ICAM1 on the capacity of the expanded cytotoxic T-cells to kill ovalbumin-expressing cancer cells. To address this question, we established a live cell, microscopy-based killing assay, testing the ability of treated OT-I T-cells to kill ovalbumin-expressing B16 melanoma cells. These target cells also express GFP, enabling automated evaluation of the number of viable cells from microscopy images. We then imaged co-cultures of the target melanoma cells with OT-I T-cells that were pre-cultured on uncoated substrates or on substrate-immobilized CCL21 + ICAM1.

**Figure 4:**
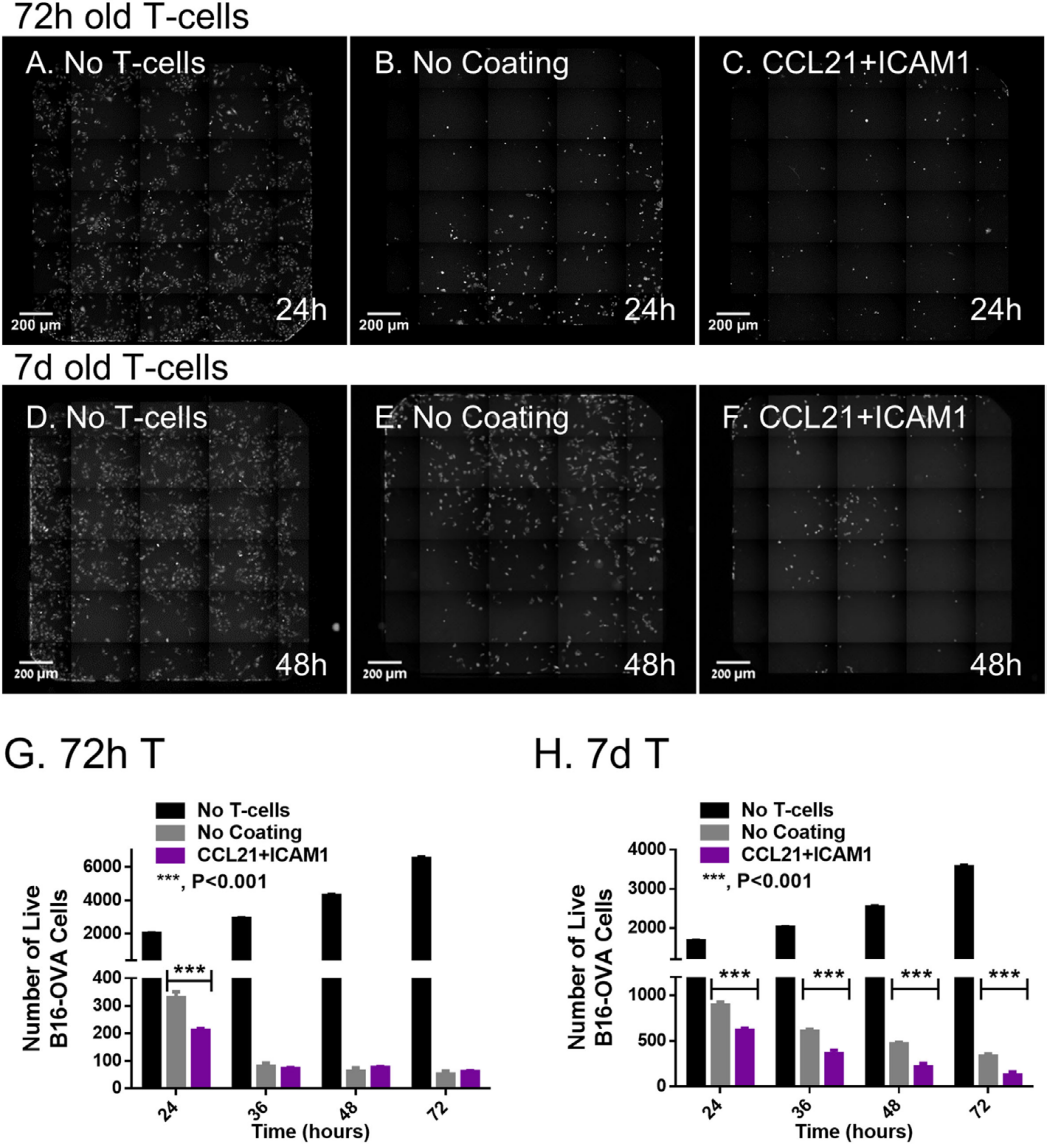
Substrate-immobilized CCL21 + intercellular adhesion molecule 1 augment the killing efficiency of cytotoxic T-cells *in vitro*. CD8^+^ T-cells, pre-cultured for 72 h **(A–C)** or 7 days **(D–F)**, were subsequently co-cultured with B16-ovalbumin-GFP cells, in a 3:1 ratio, respectively. **(A–F)** Representative fluorescence microscopy images, stitched so that each displays an entire well in a 384-well plate; live B16 cells (expressing GFP) are seen in white. **(G,H)** Bar graphs illustrating the number of viable B16-ovalbumin cells, in co-cultures with T-cells that were pre-cultured for 72 h **(G)** or 7 days **(H)**, as quantified using automated image analysis [data are from one experiment representative of at least three independent experiments with 10 replicates each (see Figure S3A in Supplementary Material). Error bars represent SEM. Calculated *p*-values (using standard *t*-test) are as indicated in the Figure]. Scale bar: 200 µm.

**Figure 5:**
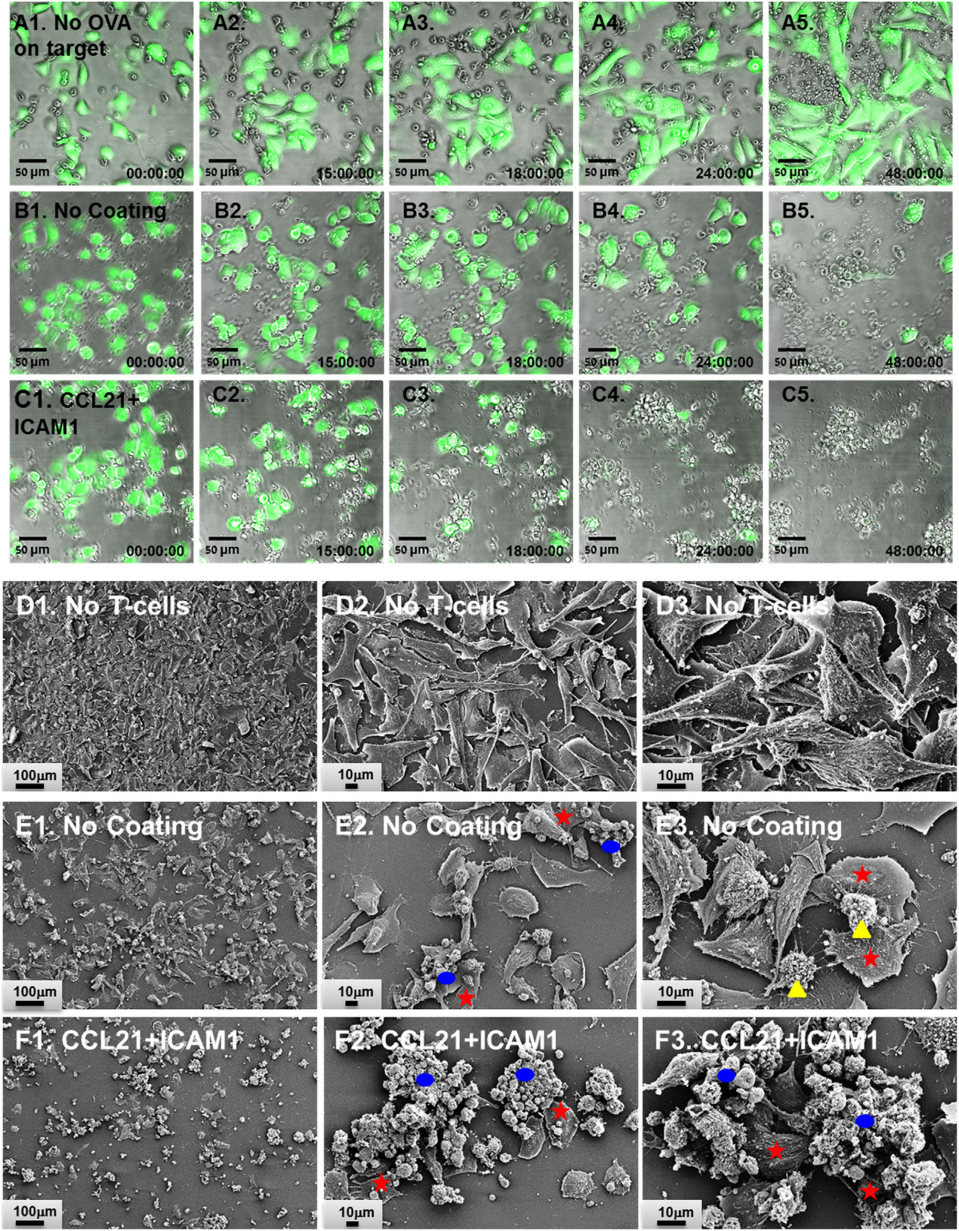
CD8^+^ T-cells pre-cultured on substrate-immobilized CCL21 + intercellular adhesion molecule 1 (ICAM1) kill target cells more rapidly. **(A1–C5)** Representative overlays of time-lapse phase contrast and florescence imaging. OT-I CD8^+^ T-cells (unstained) were pre-cultured for 7 days prior to co-culturing with target cells, on either an uncoated substrate **(A1–A5,B1–B5)** or on substrate-immobilized CCL21 + ICAM1 **(C1–C5)**. T-cells were then co-cultured with either B16-GFP cells **(A1–A5)** or B16-ovalbumin-GFP cells **(B1–C5)**. Live target cells are shown in green. B16-GFP cells, which do not express ovalbumin, were not killed by OT-I T-cells **(A1–A5)**. Substrate-immobilized CCL21 + ICAM1 induced faster killing of B16-ovalbumin-GFP cells **(C1–C5)**, compared to OT-I T-cells pre-cultured on uncoated substrates **(B1–B5)**. Time stamp: hh:mm:ss. Scale bar: 50 µm. **(D1–F3)** Representative scanning electron micrographs of B16-ovalbumin-GFP cells (large cells spread on the substrate), cultured alone **(D1–D3)**, or co-cultured for 16 h with OT-I T-cells pre-cultured for 7 days on either an uncoated substrate **(E1–E3)**, or on substrate-immobilized CCL21 + ICAM1 **(F1–F3)**. T-cells grown on substrate-immobilized CCL21 + ICAM1 **(F1–F3)** killed more target cells, as demonstrated by the lower number of remaining B16 cells. Representative target cells are denoted with red stars. Representative T-cell clusters are denoted with blue ellipsoids. Representative isolated T-cells are denoted with yellow triangles. Scale bar: In **E1, F1**—100 µm; in **E2, E3, F2**, and **F3**—10 µm.

We found that T-cells that were pre-cultured for 72 h together with ovalbumin-loaded DCs on substrate-immobilized CCL21 + ICAM1 killed target cells faster than cells that were pre-cultured on an uncoated substrate (Figures 4A–C,G). Thus, at 24 h, the number of remaining live target cells was 60% lower when using cells pre-cultured on the coated substrate (Figure 4C). When co-cultured with the target cells for longer periods (48 h or more), both T-cell populations reached a plateau at the maximal killing level (Figure 4G). We also evaluated the killing capacity of T-cells pre-cultured on substrate-immobilized CCL21 + ICAM1 for longer periods of time. A 7-day pre-culture somewhat reduced the killing capacity of the cytotoxic T-cells, regardless of the substrate on which they were growing. Yet, T-cells pre-cultured on CCL21 + ICAM still outperformed T-cells pre-cultured on uncoated substrates, killing up to 2.5 times more target cells by 24–72 h of co-culturing (Figures 4D–F,H and 5; Figure S3A and Video S1 in Supplementary Material). We note that, as shown above, longer pre-culture on the coated substrate yielded a larger number of T-cells. Thus, a balance between cell number and killing capacity may be required, based on the needs of the specific application. Time-lapse microscopy (Figures 5A1–C5) and scanning electron microscopy (Figures 5D1–F3) supported these findings, and provided direct evidence for the more rapid killing by T-cells pre-cultured on CCL21 + ICAM1-coated substrates (Figures 5C1–C5,F1–F3), compared to those pre-cultured on uncoated substrates (Figures 5B1–B5,E1–E3), demonstrated by fewer remaining target melanoma cells.

It should be noted that, in agreement with our previous findings involving CD4^+^ T-cells (28), addition of IL-6 to the culture medium of CD8^+^ T-cells adhering to substrate-immobilized CCL21 + ICAM1 further augmented their expansion (Figure S3B in Supplementary Material). However, addition of IL-6 significantly impaired the cytotoxic effectiveness of the expanded T-cells (Figure S3C and Video S1 in Supplementary Material) and was, therefore, excluded from the medium in further experiments.

**Figure 6:**
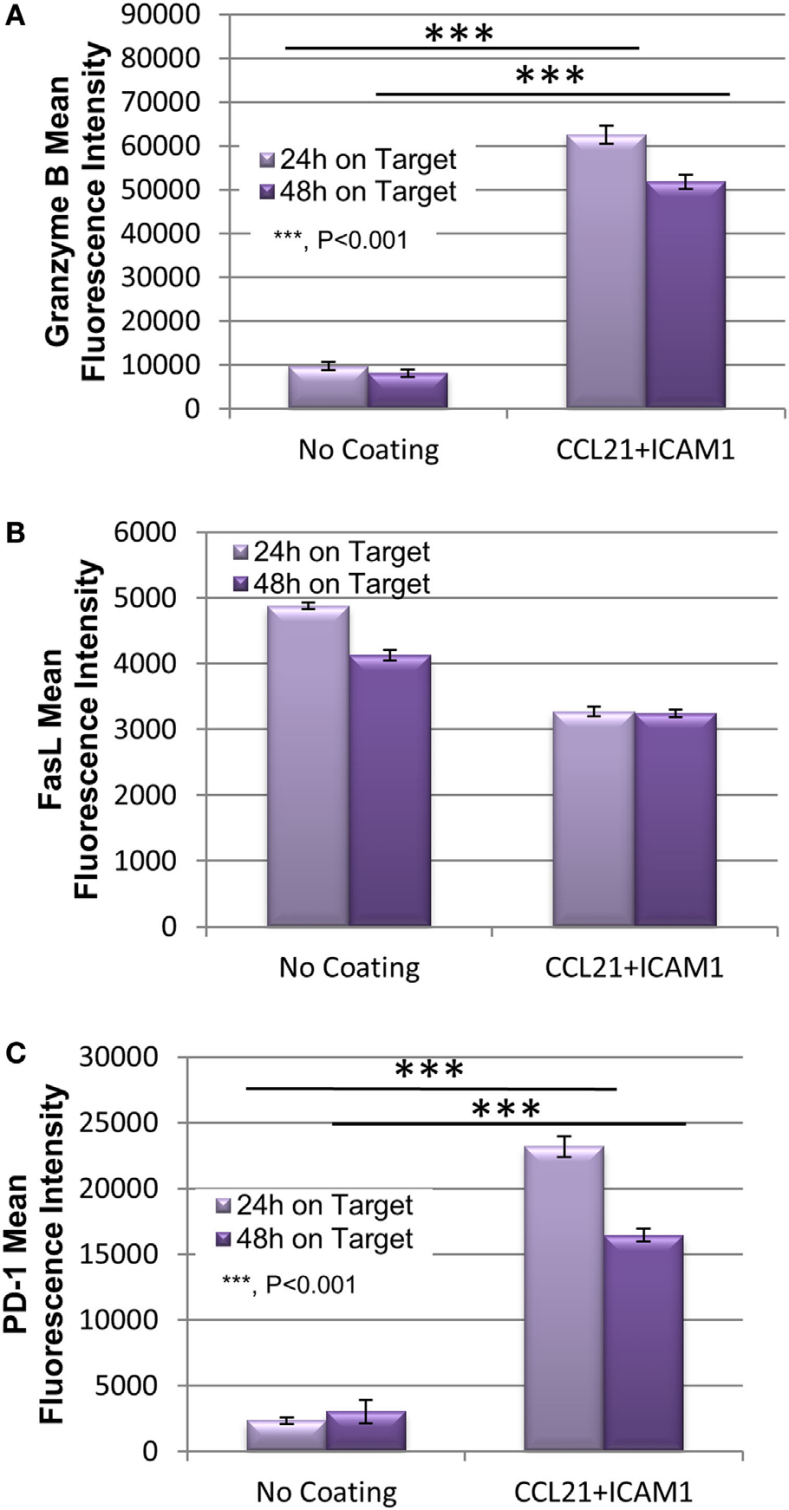
Substrate-immobilized CCL21 + ICAM1 increase T-cell expression of Granzyme B and PD-1, while not affecting FasL. **(A–C)** Bar graph illustrating the mean fluorescence intensity of granzyme B **(A)**, FasL **(B)**, and PD-1 **(C)** in cytotoxic T-cells following their incubation for 24 and 48 h with B16-ovalbumin target cells. T-cells were pre-cultured for 7 days on either an uncoated substrate, or on substrate-immobilized CCL21 + ICAM1 [data are from one experiment representative of three independent experiments with four replicates each (see Figure S4 in Supplementary Material). Error bars represent SEM. Calculated *p*-values (using standard *t*-test) are as indicated in the Figure].

To explore the mechanism underlying the enhanced killing efficiency induced by the SIN, we quantified the expression of the key killing mediators granzyme B and FasL, as well as the exhaustion marker PD-1. The levels of these proteins in OT-I T-cells were measured using flow cytometry after 24 or 48 h of co-culture with target B16 cells. As shown, CD8^+^ T-cells pre- cultured on CCL21 + ICAM1-coated substrates displayed a 6.5 higher level of granzyme B, compared with cells pre-cultured on an uncoated substrate (Figure 6A; Figure S4 in Supplementary Material), whereas no significant difference was evident in the level of expression of FasL (Figure 6B; Figure S4 in Supplementary Material), which mediates killing in a different pathway. The expression of PD-1, a key T-cell exhaustion marker, was also elevated by CCL21 + ICAM1, by up to 10-fold (Figure 6C; Figure S4 in Supplementary Material).

### Substrate-Immobilized CCL21 + ICAM1 Augment *In Vivo* Tumor Suppression by Adoptively Transferred CD8^+^ T-Cells

**Figure 7:**
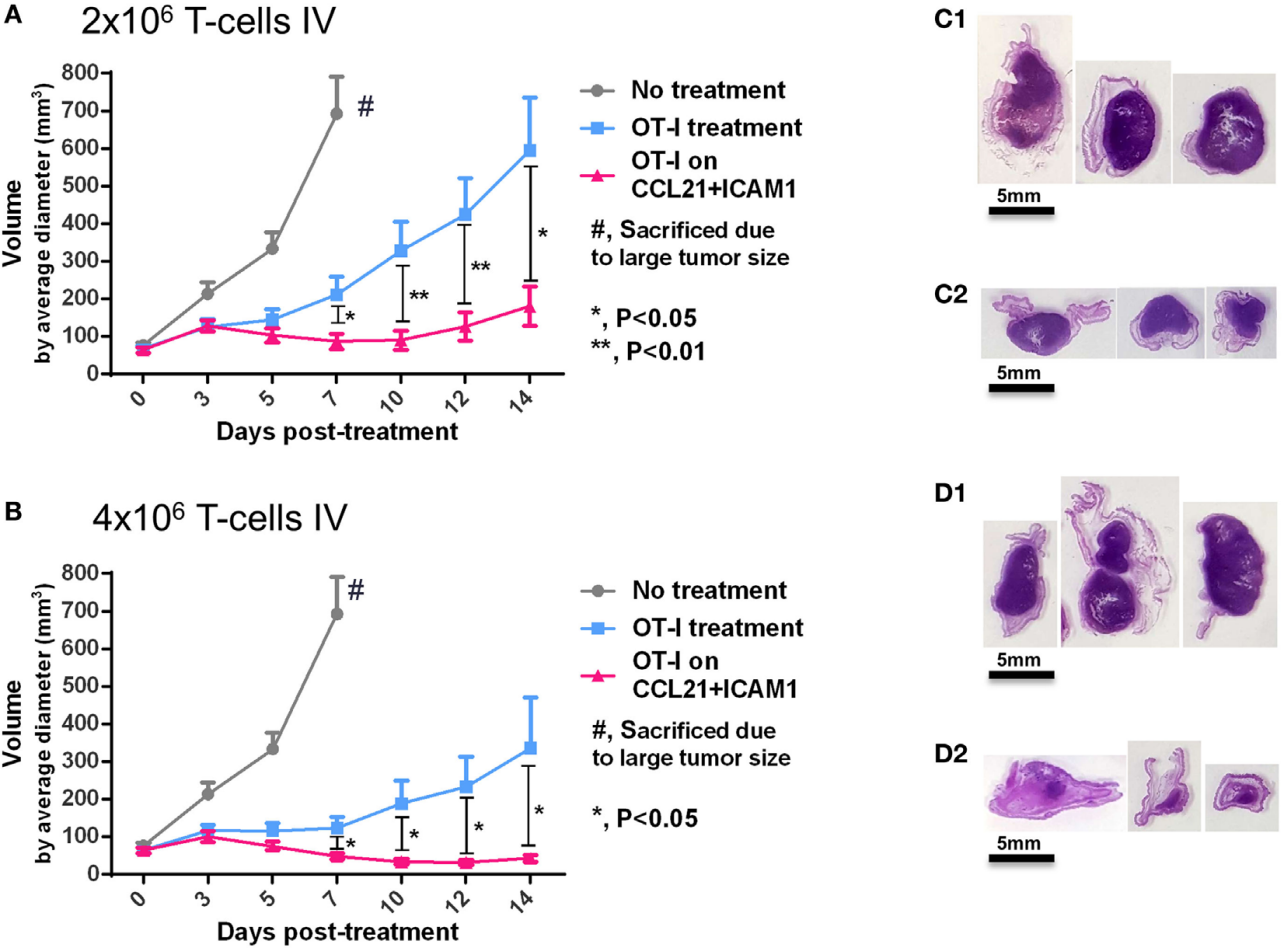
Substrate-immobilized CCL21 + intercellular adhesion molecule 1 (ICAM1) augment tumor suppression by CD8^+^ T-cells *in vivo*. **(A,B)** C57BL/6 mice were injected with 2 × 10^6^ B16 cells expressing ovalbumin and GFP. Seven days later, tumor-bearing mice were split into treatment groups, according to similar tumor average size and distribution, and injected intravenously with 2 × 10^6^ **(A)** or 4 × 10^6^ activated OT-I T-cells **(B)**. Averages of measured tumor volumes are shown for either untreated mice (gray), or for mice treated with OT-I T-cells pre-cultured for 7 days on an uncoated substrate (blue), or on substrate-immobilized CCL21 + ICAM1 (pink). **(C1–D2)** Images of histology sections of three representative tumors of mice treated with 2 × 10^6^ OT-I T-cells pre-cultured on uncoated substrates **(D1)**, or on substrate-immobilized CCL21 + ICAM1 **(D2)**; or treated with 4 × 10^6^ OT-I T-cells pre-cultured on uncoated substrates **(E1)**, or on substrate-immobilized CCL21 + ICAM1 coating **(E2)**. Scale bar: 5 mm. T-cells pre-cultured on substrate-immobilized CCL21 + ICAM1 suppressed tumor growth to a significantly greater extent than T-cells cultured on uncoated substrates. *N* = 10 mice per group. Error bars represent SEM.

To assess the capacity of CCL21 + ICAM1-stimulated CD8^+^ T-cells to suppress tumor development *in vivo*, we treated mice bearing B16-ovalbumin tumors with T-cells that were pre-cultured for 7 days on either uncoated substrates, or on substrate-immobilized CCL21 + ICAM1. Our *in vivo* findings (Figure 7) correlated with those found *in vitro* (Figures 4–6) and show that OT-I T-cells pre-cultured on substrate-immobilized CCL21 + ICAM1, suppressed tumor growth to a significantly greater extent than those pre-cultured on an uncoated substrate (Figures 7A,B).

This effect was dose-dependent, leading to a greater than threefold reduction in average tumor volume (at the experimental end point of 14 days), in the case of transferring 2 × 10^6^ T-cells (Figure 7A), and an eightfold reduction in the case of transferring 4 × 10^6^ T-cells (Figure 7B). Notably, the tumor-growing kinetics also differed between treatment groups. While tumors treated with T-cells pre-cultured on uncoated substrates continuously grew in size over time, the tumors treated with T-cells cultured on CCL21 + ICAM1 substrates displayed almost no increase in tumor size, throughout the follow-up period (14 days) (Figures 7A,B).

## Discussion

In this study, we developed a highly potent molecular microenvironment, inspired by the molecular organization of the natural immune niche in the lymph node, for the efficient *ex vivo* expansion and functional reinforcement of antigen-specific CD8^+^ T-cells. Our efforts were primarily directed toward enhancement of the cytotoxic reactivity of T-lymphocytes against cancerous cells. We show that exposure of CD8^+^ T-cells to substrate-immobilized CCL21 [a potent chemokine with multiple effects on T-cell migration, recruitment, and activation (25, 30–32, 33)] and ICAM1 [an adhesion molecule that activates LFA1-mediated signaling (22, 29)] induces a dramatic shift in T-cell organization, accompanied by major enhancement of their proliferation, intrinsic killing efficiency in culture, and tumor suppression *in vivo*.

Our findings demonstrate that culturing cytotoxic T-cells on substrate-immobilized CCL21 increases the size of T-cell clusters (Figures 1A1–B2). These large clusters could be attributed to LFA1 activation (22, 29), chemokine enhanced co-stimulation or proliferation (41), or to the enhanced cell migration induced by CCL21 (25, 32). Clustering can enhance T-cell–DC interactions, as well as promote paracrine stimulation by neighboring T-cells within the same cluster (17, 42–44). However, formation of large clusters, *per se*, could act as a “double-edged sword” by limiting access to soluble factors, and promoting potential inhibitory signals (45). Since clustering was previously reported to limit cytotoxic T-cell function (45), we hypothesized that CD8^+^ T-cells might benefit from an alternative activation route, mediated by substrate-immobilized ICAM1, which might compete with cluster formation. Similarly to our previous findings with CD4^+^ T-cells (28), substrate-immobilized ICAM1, with or without CCL21, indeed prevents the formation of large clusters, and induces cell spreading of CD8^+^ T-cells on the tissue culture substrate (Figures 1C1,D1).

We further found that the combination of substrate-immobilized CCL21 and ICAM1 collectively increases CD8^+^ T-cell expansion by enhancing cell proliferation (Figures 2E,F), rather than by reducing cell death (Figures 2G,H). This could be attributed to ICAM1-mediated stimulation of LFA1, while avoiding the inhibitory effect observed in the large 3D clusters, and reinforcing and prolonging interaction with the substrate-immobilized CCL21. Notably, our previous findings indicated that the increase in T-cell expansion is not merely the result of the physical tethering of LFA1 to the substrate (e.g., by substrate-boundanti-LFA1 antibodies), but results from specific ICAM1-dependent signaling (28). Activation of T-cells with microbeads coated with anti-CD3 + anti-CD28 antibodies, on substrates coated with CCL21 + ICAM1, also significantly augmented T-cell proliferation and yield (Figures 3A–C,F,G), indicating that the benefits of this coating combination are also relevant to other forms of T cell activation.

Since T-cell survival is often supported by paracrine signals from adjacent T-cells, the expansion of low T-cell concentrations is challenging. It is, therefore, noteworthy that substrate-immobilized CCL21 + ICAM1 displayed pronounced enhancement of cell expansion, even when the number of seeded CD8^+^ T-cells was extremely low (see Figure S2 in Supplementary Material). This remarkable increase in seeding efficiency could be helpful in cases where only a low number of cytotoxic T-cells are available; for example, when a small number of tumor-infiltrating lymphocytes are harvested from a tumor for adoptive cell therapy (46, 47).

Despite the importance of efficient T-cell expansion and yield, verifying cell functionality is crucial, since expanded T-cells might display impaired cytotoxicity as a result of anergy (7) or exhaustion (8, 9). In light of these possible dysfunctions, we directly tested the intrinsic cytotoxic functionality of CD8^+^ T-cells cultured on the different substrates, using live-cell imaging. Notably, we found that CD8^+^ T-cells pre-cultured on substrate-immobilized CCL21 + ICAM1 killed target cells significantly faster than those cultured on an uncoated substrate (Figures 4 and 5; Video S1 in Supplementary Material). The improvement in killing time was more pronounced when cells were pre-cultured for longer periods (Figure 4, 7 vs. 3 days).

Our results show that the signals provided by the CCL21 + ICAM1 coating are not required during the killing phase, as efficient killing was obtained on uncoated substrates. This could indicate that there is no need for continuous stimulation by CCL21 + ICAM1 at the target site for the beneficial effect of their potentiated killing to persist, further supporting their use in *ex vivo* manipulation for adoptive cell therapy.

In addition to CCL21 and ICAM1, we also examined whether supplementation with various cytokines (IL-2, IL-12, IL-6, IL-7, IL-15, and IFNγ) could further enhance cell yield (data not shown). In accordance with our previous findings with CD4^+^ T-cells (28), we found that addition of IL-6, a known supporter of T-cell survival (38, 39), collectively with substrate-immobilized CCL21 + ICAM1, increased CD8^+^ T-cell expansion by ~1.5-fold (Figure S3B in Supplementary Material). However, the addition of IL-6 significantly attenuated T-cell killing of target cells (Figure S3C and Video S1 in Supplementary Material). These findings indicate that increased cell yield, or high cell survival rate, do not, in and of themselves, constitute direct indicators of T-cell functionality, which should be verified independently.

Further insights into the mechanism underlying the increase in the cytotoxic potency of CD8^+^ T-cells following treatment with CCL21 + ICAM1, was obtained from quantification of the classical components of the killing machinery; namely, granzyme B, and FasL (Figure 6). We found that the expression of granzyme B was elevated in OT-I T-cells that were activated by CCL21 + ICAM1, compared with those cultured on uncoated dishes (Figure 6A; Figure S4 in Supplementary Material). Since granzyme B is a main mediator of T-cell cytotoxicity (48–50), its increased expression could account for augmented killing capacity. No significant change was evident in the expression level of FasL (Figure 6B; Figure S4 in Supplementary Material), which mediates an alternate killing pathway (51, 52).

The expression of PD-1, a known T-cell exhaustion marker (8, 9) that is often exploited by tumors for immune evasion (53, 54), was also elevated in T-cells cultured on CCL21 + ICAM1 substrates (Figure 6C; Figure S2 in Supplementary Material). This elevation could potentially impair T-cell mediated killing, especially of tumor cells expressing PD-1 ligand. Interestingly, despite the known expression of PD-1 ligand on B16 cells (55), their killing by T-cells pre-cultured on CCL21 + ICAM1 substrates was significantly faster (Figures 4 and 5). Since a strong TCR stimulation combined with co-stimulation by CD28 (56) or IL-2 (57) were shown to overcome the inhibitory effect of PD-1 binding, it is possible that CCL21 + ICAM1 signaling has a similar effect.

While the enhanced cytotoxicity of T-cells cultured on CCL21 + ICAM1 toward cultured cancer cells was compelling, we further tested their capacity to kill tumor cells *in vivo*. In this setting, additional physiological factors, which alter the functionality of T-cells, come into effect. Among these are the interplay with other types of regulatory systems, immune and stromal, anatomical and cellular barriers and, often, the development of an immunosuppressive microenvironment involving, for instance, the PD-1 receptor (53).

Direct *in vivo* testing indicated that CD8^+^ T-cells pre-cultured for 7 days on substrate-immobilized CCL21 + ICAM1 suppressed tumor growth to a significantly greater extent than T-cells cultured on unmodified substrates (Figure 7). This tumor suppression correlated with the number of adoptively transferred T-cells (Figures 7A,D2). Most importantly, tumor suppression by T-cells pre-cultured on CCL21 + ICAM1 persisted throughout the 14-day experiment, with almost no increase in tumor size over time, compared to the constantly growing tumors treated with T-cells pre-cultured on uncoated substrates (Figures 7A,C).

In conclusion, we showed that pre-culturing CD8^+^ T-cells on substrate-immobilized CCL21 + ICAM1 increases both the proliferation of T-cells and their tumor-killing capacity, leading to enhanced tumor suppression abilities. Our findings demonstrate the power of a SIN to regulate cell behavior and fate. Such SINs can enable a deeper exploration of the molecular mechanisms affecting immune cell interactions, and can be utilized for the *ex vivo* culture of T-cells with improved function, for adoptive immunotherapy.

## Ethics Statement

C57BL/6 mice were obtained from Harlan Laboratories (Rehovot, Israel), and OT-I mice from Jackson Laboratories (Bar Harbor, ME, USA). All mice were 5–12 weeks old. Mice were maintained at the Weizmann Institute’s Lorry Lokey Pre-Clinical Research Facility and cared for in accordance with national and institutional guidelines. Experiments were approved by the Institutional Animal Care and Use Committee.

## Author Contributions

SA-L participated in planning the experiments, their execution, data analysis, and manuscript preparation. BG and NF participated in planning the experiments, data analysis, and manuscript preparation.

## Conflict of Interest Statement

The authors declare that the research was conducted in the absence of any commercial or financial relationships that could be construed as a potential conflict of interest.

## Supplementary Material

Figure S1 Substrate-immobilized CCL21 + intercellular adhesion molecule 1 (ICAM1) increase cytotoxic T-cell number and proliferation. **(A)** Relative fold change in live T-cell numbers, measured in all independent experiments, using either metabolic or microscopy-based assays (see “Materials and Methods”). Each symbol denotes the expansion of cells growing on CCL21 + ICAM1 (i.e., final/initial cell number, averaged over 3–10 replicates that were performed at the same time and under the same conditions) normalized to that of cells growing on uncoated surfaces, in one independent experiment (i.e., with the same initial cell number, incubation time and plate format, and using the same cell enumeration method). Seeded cell numbers ranged from 1,500 to 60,000 per well of 384- or 96-well plate. Incubation times were 72 h (left) or 5–7 days (right). Error bars represent SEM. **(B)** Similar to panel **(A)**, showing the relative fold change in cell proliferation (measured using CFSE mean fluorescent intensity), for cells growing on CCL21 + ICAM1, normalized to that of cells growing on uncoated surfaces. Results are shown for activation of OT-I T cells with OVA loaded dendritic cells (left) or for activation with microbeads coated with anti-CD3/anti-CD28 antibodies (right).

Figure S2 Substrate-immobilized CCL21 + intercellular adhesion molecule 1 (ICAM1) increase the culture density of T-cells seeded at low cell concentrations. Representative stitched fluorescence images of entire 384-wells seeded with low concentrations of T-cells grown on either uncoated substrates **(A,C)** or on CCL21 + ICAM1 substrates **(B,D)**, for 72 h. Stained nuclei are seen in white. Scale bar: 100 µm. Concentrations of seeded cells were either 7,500/ml **(A,B)** (750 cells/well) or 3,250/ml **(C,D)** (325 cells/well). On uncoated substrates, final T-cell density was low, as demonstrated by the scarcity of fluorescently stained cells **(A)**, with ~2,650 cells per well and **(C)**, with ~1,365 cells per well, while on substrate-immobilized CCL21 + ICAM1 substrates, T-cell density was increased **(B)**, with ~16,810 cells per well, and **(D)** with ~4,430 cells per well. We note cells in large clusters cannot be reliably counted as they are out of the focal plane, due to the 3-dimensional nature of cell clusters. However, as there are more large cell clusters on the coated surface, the actual differences in cell numbers between the coated and uncoated surfaces are larger.

Figure S3 Substrate-immobilized CCL21 + intercellular adhesion molecule 1 (ICAM1) augment the killing efficiency of cytotoxic T-cells while combining IL-6 further increases viable T-cell numbers, yet attenuates their killing efficiency. **(A)** Fold change in the average number of live B16-ovalbumin-GFP cells, co-cultured for 72 h with T-cells that were pre-cultured for 7 days. Cells were seeded at a ratio of 1/3-3 T-cells per target cell. Each symbol denotes the fold change of the uncoated group normalized to that of the CCL21 + ICAM1group in one independent experiment: average of 5–10 replicates that were performed at the same date and with the same conditions (same initial cell number, incubation time and format, and cell enumeration method). Error bars represent SEM. **(B)** Bar graphs illustrating the number of viable T-cells cultured on CCL2 + ICAM1 substrates, normalized to those in a control group, cultured on substrates with no coating and no IL-6, quantified using automated image analysis. Data are representative of at least three independent experiments with 20 replicates each. Error bars represent SEM. Calculated *p*-values (using standard *t*-test) are as indicated in the Figure. Number of T-cells seeded per well: 3 × 10^3^. **(C)** Bar graphs illustrating the number of viable B16-ovalbumin cells, co-cultured with T-cells pre-cultured for 7 days, quantified using automated image analysis. Data are representative of at least three independent experiments with 10 replicates each. Error bars represent SEM. Calculated *p*-values (standard *t*-test) are as indicated in the Figure. CCL21 + ICAM1 augment the killing of target cells by T-cells pre-cultured for 7 days, while addition of soluble IL-6 to the substrate-immobilized CCL21 + ICAM1 attenuates it.

Figure S4 Substrate-immobilized CCL21 + intercellular adhesion molecule 1 (ICAM1) increase T-cell expression of Granzyme B and PD-1, while not affecting FasL. Fold change in the mean fluorescence intensity of Granzyme B, FasL, and PD-1. The mean fluorescence intensity in cells cultured on CCL21 + ICAM1-coated substrates, measured at the experimental end point, was normalized to that obtained on uncoated substrates. Each symbol denotes the average fluorescent intensity of the CCL21 + ICAM1group normalized to that of the uncoated group in one independent experiment: average of four replicates that were performed at the same date and with the same conditions (same initial cell number, incubation time and format, and cell enumeration method). Error bars represent SEM.

Video S1 Culturing OT-I CD8^+^ cytotoxic T-cells for 7 days on substrate-immobilized CCL21 + intercellular adhesion molecule 1 (ICAM1) reduces the killing time of ovalbumin-expressing B16 cells, while addition of soluble IL-6 to the culture increases it. CD8^+^ T-cells (unstained), were pre-cultured for 7 days prior to co-culturing, on either uncoated substrates **(A,B)**, or on substrate-immobilized CCL21 + ICAM1 **(C,D)**, with **(D)** or without **(C)** the addition of soluble IL-6. T-cells were then co-cultured with either B16-GFP cells **(A)** or B16-ovalbumin-GFP cells **(B–D)**, live cells shown in green. The killing of live (e.g., GFP-expressing) B16 cells is noticeable when the dead cells stop expressing the GFP color, and turn from green-colored to non-colored as they detach from the substrate. OT-I cytotoxic T-cells pre-cultured on substrate-immobilized CCL21 + ICAM1 **(B)** killed ovalbumin-expressing B16 significantly faster than those cultured on an uncoated substrate **(A)**, as demonstrated by the earlier disappearance of green cells (see quantification in Figure 4). However, OT-I cytotoxic T-cells pre-cultured with the addition of soluble IL-6 on top of the substrate-immobilized CCL21 + ICAM1, demonstrated a much slower rate of killing of B16 target cells **(C)** than those cultured on an uncoated substrate **(A)**. No cell killing was evident when OT-I T-cells were co-cultured with B16-GFP cells, which did not express the ovalbumin peptide **(D)**. Time stamp, hh:mm:ss. Scale bar: 50 µm.
