# Supplementary figures and images for: Expansion and Antitumor Cytotoxicity of T-Cells Are Augmented by Substrate-Bound CCL21 and Intercellular Adhesion Molecule 1
Source: Front Immunol. 2018 Jun 11;9:1303. doi: 10.3389/fimmu.2018.01303 (PMC6004589; doi:10.3389/fimmu.2018.01303)

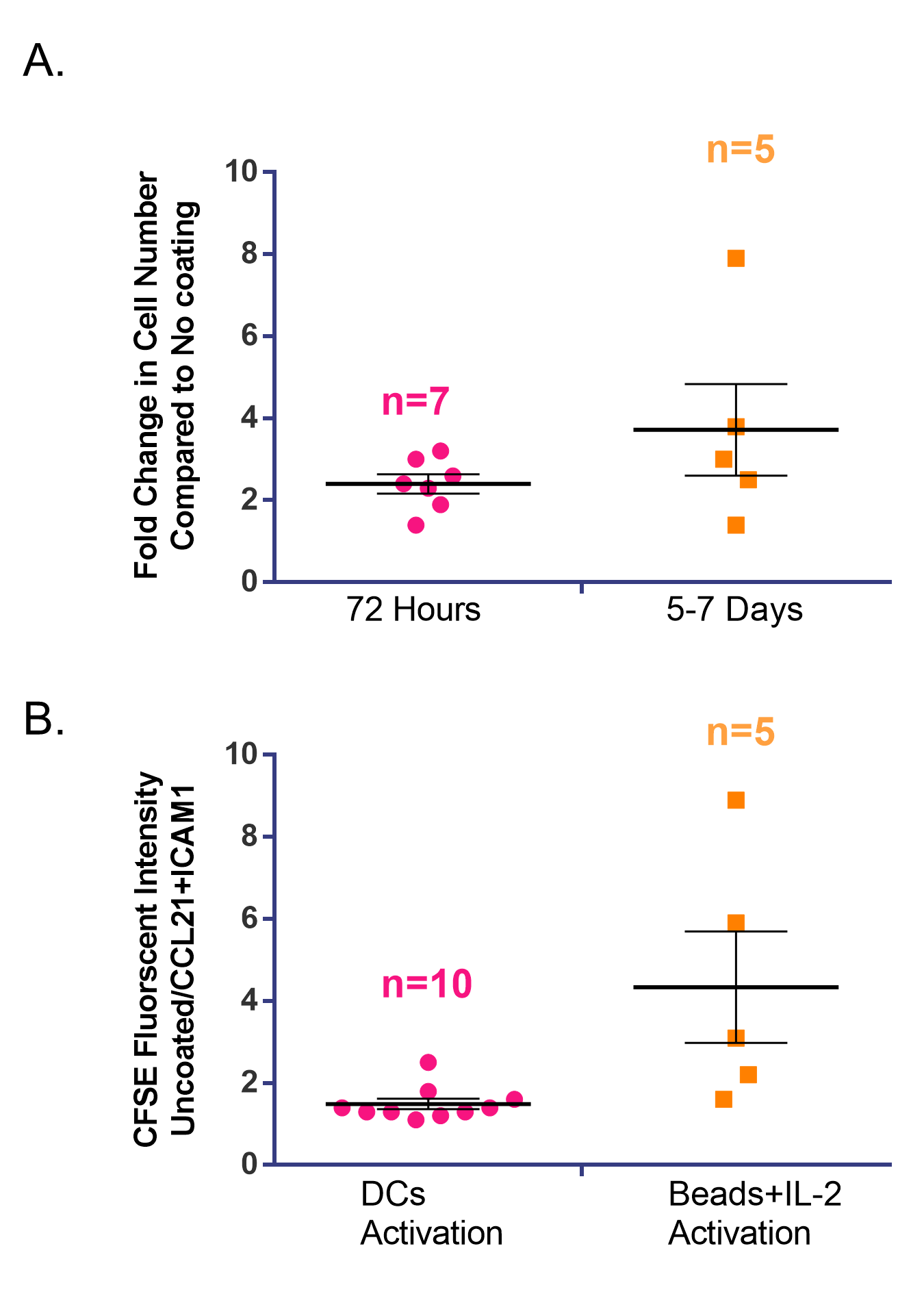

Supplement: Figure S1 — Substrate-immobilized CCL21 + intercellular adhesion molecule 1 (ICAM1) increase cytotoxic T-cell number and proliferation. (A) Relative fold change in live T-cell numbers, measured in all independent experiments, using either metabolic or microscopy-based assays (see “Materials and Methods”). Each symbol denotes the expansion of cells growing on CCL21 + ICAM1 (i.e., final/initial cell number, averaged over 3–10 replicates that were performed at the same time and under the same conditions) normalized to that of cells growing on uncoated surfaces, in one independent experiment (i.e., with the same initial cell number, incubation time and plate format, and using the same cell enumeration method). Seeded cell numbers ranged from 1,500 to 60,000 per well of 384- or 96-well plate. Incubation times were 72 h (left) or 5–7 days (right). Error bars represent SEM. (B) Similar to panel (A), showing the relative fold change in cell proliferation (measured using CFSE mean fluorescent intensity), for cells growing on CCL21 + ICAM1, normalized to that of cells growing on uncoated surfaces. Results are shown for activation of OT-I T cells with OVA loaded dendritic cells (left) or for activation with microbeads coated with anti-CD3/anti-CD28 antibodies (right). [file image_1.tif]

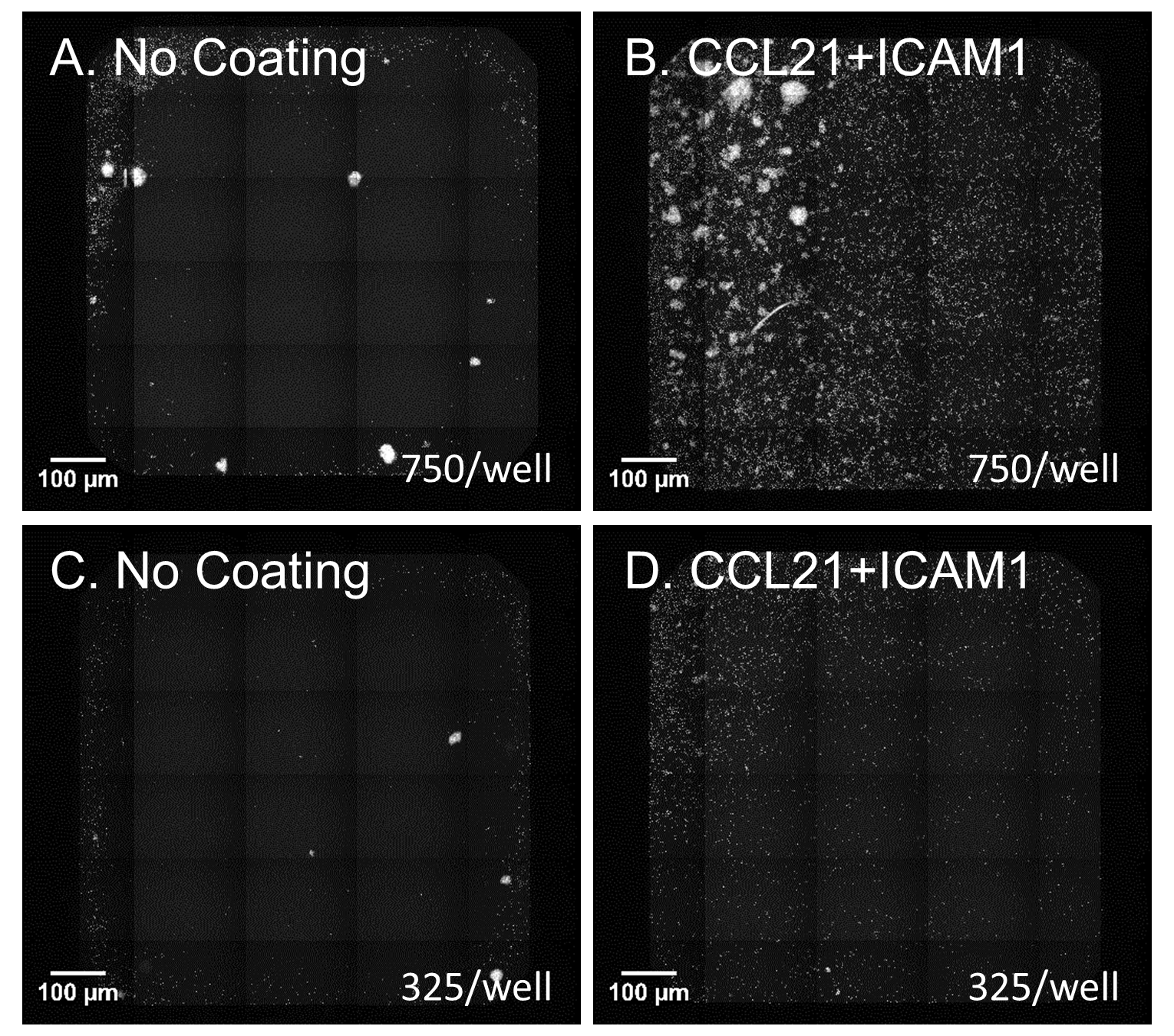

Supplement: Figure S2 — Substrate-immobilized CCL21 + intercellular adhesion molecule 1 (ICAM1) increase the culture density of T-cells seeded at low cell concentrations. Representative stitched fluorescence images of entire 384-wells seeded with low concentrations of T-cells grown on either uncoated substrates (A,C) or on CCL21 + ICAM1 substrates (B,D), for 72 h. Stained nuclei are seen in white. Scale bar: 100 µm. Concentrations of seeded cells were either 7,500/ml (A,B) (750 cells/well) or 3,250/ml (C,D) (325 cells/well). On uncoated substrates, final T-cell density was low, as demonstrated by the scarcity of fluorescently stained cells (A), with ~2,650 cells per well and (C), with ~1,365 cells per well, while on substrate-immobilized CCL21 + ICAM1 substrates, T-cell density was increased (B), with ~16,810 cells per well, and (D) with ~4,430 cells per well. We note cells in large clusters cannot be reliably counted as they are out of the focal plane, due to the 3-dimensional nature of cell clusters. However, as there are more large cell clusters on the coated surface, the actual differences in cell numbers between the coated and uncoated surfaces are larger. [file image_2.tif]

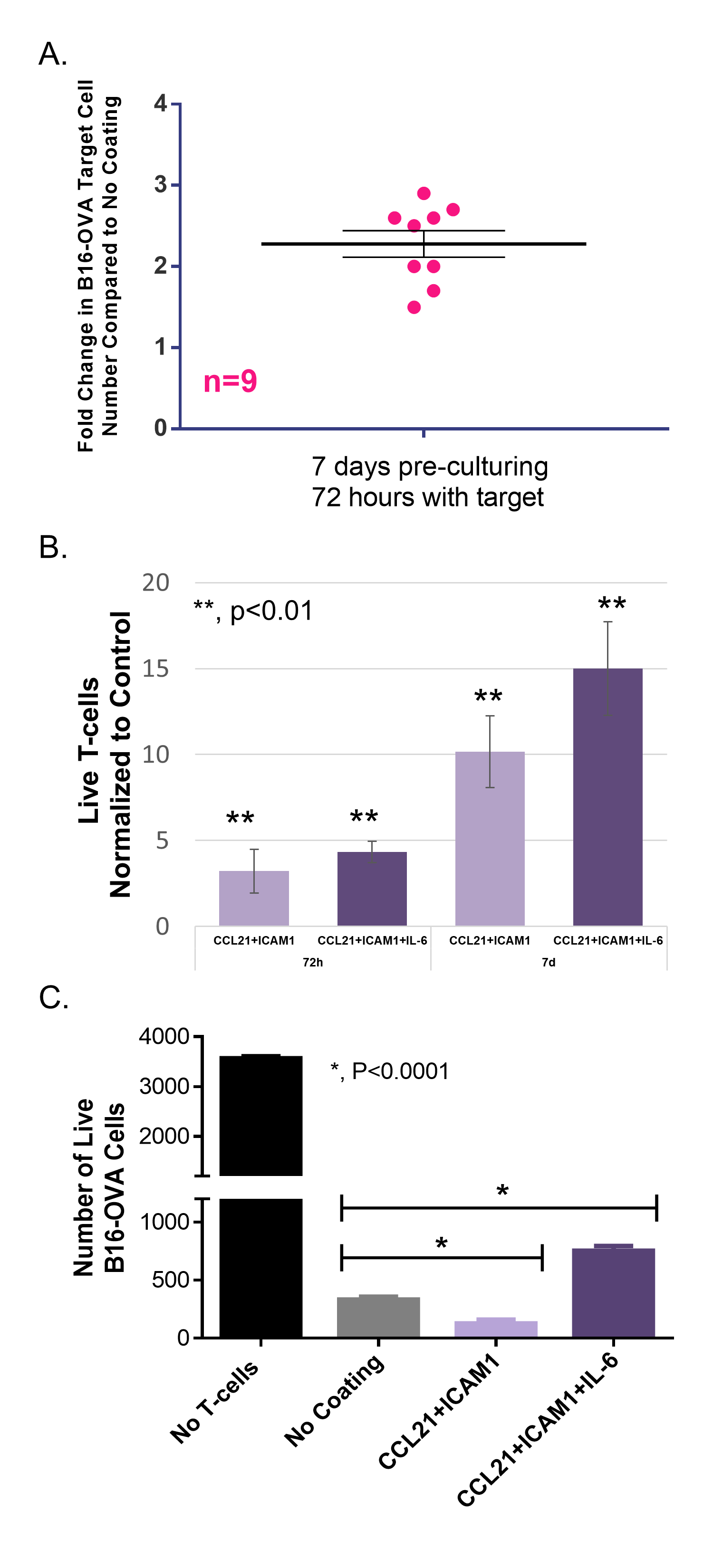

Supplement: Figure S3 — Substrate-immobilized CCL21 + intercellular adhesion molecule 1 (ICAM1) augment the killing efficiency of cytotoxic T-cells while combining IL-6 further increases viable T-cell numbers, yet attenuates their killing efficiency. (A) Fold change in the average number of live B16-ovalbumin-GFP cells, co-cultured for 72 h with T-cells that were pre-cultured for 7 days. Cells were seeded at a ratio of 1/3-3 T-cells per target cell. Each symbol denotes the fold change of the uncoated group normalized to that of the CCL21 + ICAM1group in one independent experiment: average of 5–10 replicates that were performed at the same date and with the same conditions (same initial cell number, incubation time and format, and cell enumeration method). Error bars represent SEM. (B) Bar graphs illustrating the number of viable T-cells cultured on CCL2 + ICAM1 substrates, normalized to those in a control group, cultured on substrates with no coating and no IL-6, quantified using automated image analysis. Data are representative of at least three independent experiments with 20 replicates each. Error bars represent SEM. Calculated p-values (using standard t-test) are as indicated in the Figure. Number of T-cells seeded per well: 3 × 10^3^. (C) Bar graphs illustrating the number of viable B16-ovalbumin cells, co-cultured with T-cells pre-cultured for 7 days, quantified using automated image analysis. Data are representative of at least three independent experiments with 10 replicates each. Error bars represent SEM. Calculated p-values (standard t-test) are as indicated in the Figure. CCL21 + ICAM1 augment the killing of target cells by T-cells pre-cultured for 7 days, while addition of soluble IL-6 to the substrate-immobilized CCL21 + ICAM1 attenuates it. [file image_3.tif]

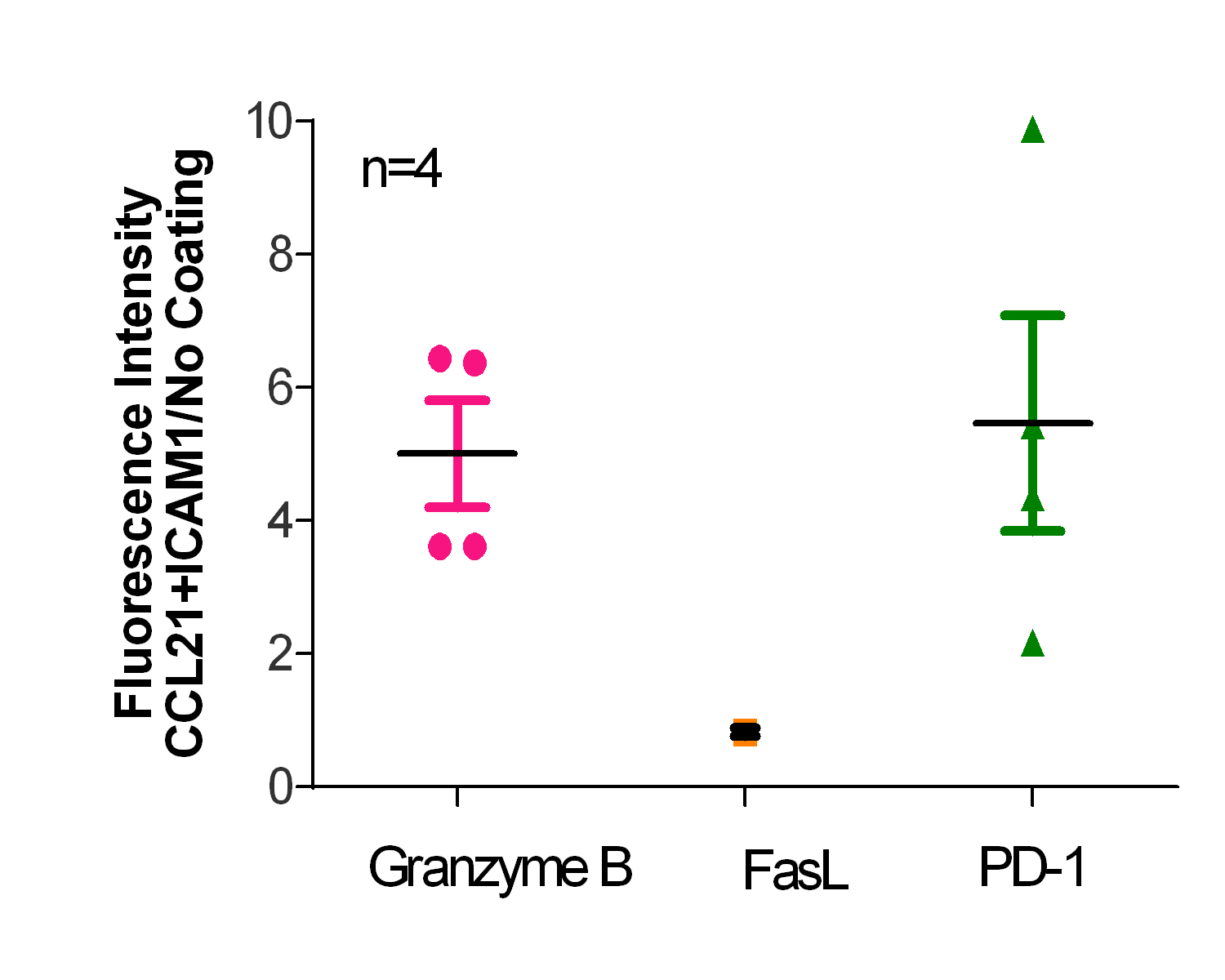

Supplement: Figure S4 — Substrate-immobilized CCL21 + intercellular adhesion molecule 1 (ICAM1) increase T-cell expression of Granzyme B and PD-1, while not affecting FasL. Fold change in the mean fluorescence intensity of Granzyme B, FasL, and PD-1. The mean fluorescence intensity in cells cultured on CCL21 + ICAM1-coated substrates, measured at the experimental end point, was normalized to that obtained on uncoated substrates. Each symbol denotes the average fluorescent intensity of the CCL21 + ICAM1group normalized to that of the uncoated group in one independent experiment: average of four replicates that were performed at the same date and with the same conditions (same initial cell number, incubation time and format, and cell enumeration method). Error bars represent SEM. [file image_4.tif]
